# Insulin potentiates lipopolysaccharide-induced IL-6 expression through epigenetic remodeling in adipocytes: *in vitro* and *in vivo* mechanistic study

**DOI:** 10.3389/fimmu.2026.1840850

**Published:** 2026-07-08

**Authors:** Fatemah Bahman, Areej Al-Roub, Nadeem Akther, Ashraf Al Madhoun, Fahd Al-Mulla, Rasheed Ahmad

**Affiliations:** 1 Immunology & Microbiology Department, Dasman Diabetes Institute, Dasman, Kuwait; 2 Translational Research Department, Dasman Diabetes Institute, Dasman, Kuwait

**Keywords:** acetylation, adipocytes, H3K9, IL-6, macrophages

## Abstract

Interleukin-6 (IL-6) is a central mediator of chronic low-grade inflammation associated with metabolic disease. Because obesity is characterized by elevated circulating insulin and metabolic endotoxemia, we investigated whether insulin modulates lipopolysaccharide (LPS) induced IL-6 expression in adipocytes and examined the underlying epigenetic mechanisms. Insulin priming markedly enhanced LPS-induced *Il6* mRNA expression (25.33 ± 0.833-fold) and protein levels (181.8 ± 2.754 pg/ml) in 3T3-L1 mouse adipocytes. Similar synergistic effects were observed in primary mouse (*Il6* mRNA; 1.364 ± 0.287-fold and protein; 298.6 ± 13.79-pg/ml) and human adipocytes (*Il6* mRNA; 12.99 ± 0.912-fold and protein; 1441 ± 68.69-pg/ml). *In vivo*, mice treated with insulin followed by LPS exposure exhibited significantly higher *Il6* expression in peripheral blood mononuclear cells and adipose tissue compared to either treatment alone. Pharmacological inhibition of PI3K signaling suppressed this effect and AKT phosphorylation. Mechanistically, epigenetic profiling revealed that insulin increased histone H3 lysine 9 acetylation (H3K9ac), an active chromatin marker, in a PI3K-dependent manner. Chromatin immunoprecipitation-quantitative polymerase chain reaction (ChIP–qPCR) analysis demonstrated an enhanced H3K9 acetylation at the NF-κB and CREB loci at the distal region and CREB/NF-IL6 locus at the proximal region of the *Il6* promoter following combined insulin and LPS stimulation; this effect was significantly attenuated upon blockade of insulin signaling. This synergistic induction was dependent on H3K9 acetylation, indicating that metabolic and inflammatory signals converge at the *Il6* promoter to promote chromatin remodeling and transcriptional co-activator recruitment. Collectively, these findings demonstrate that insulin synergizes with LPS to amplify IL-6 mediated inflammation in adipocytes through epigenetic remodeling of the *Il6* locus, linking hyperinsulinemia to chronic inflammation in obesity and insulin resistance.

## Graphical Abstract

**Figure:**
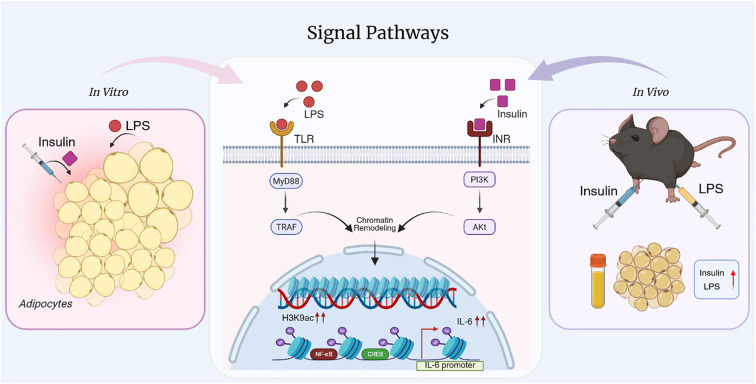

## Introduction

Obesity and type 2 diabetes are characterized by chronic low-grade inflammation that disrupts metabolic homeostasis and accelerates disease development (1–3). Adipose tissue is a central driver of metabolic inflammation, characterized by higher proinflammatory cytokine production, including IL-6, IL-1β, TNF-α and macrophage polarization toward an M1 phenotype (4, 5). The obesity-associated metabolic inflammation factors remain undefined. One important contributor is increased systemic exposure to bacterial lipopolysaccharide (LPS) resulting from impaired gut barrier function, a condition commonly referred to as metabolic endotoxemia. High-fat dietary intake impairs this process by elevating circulating LPS levels, thereby enhancing inflammatory signaling (6–8). LPS primarily activates Toll-like receptor 4 (TLR4) on macrophages, leading to the induction of proinflammatory cytokines, including IL-6 and TNF-α, which are known to disrupt insulin signaling (9, 10).

Insulin is a central regulator of glucose metabolism and has also been implicated in modulating immune responses. Several studies have reported immunomodulatory effects of insulin on cytokine production of human monocyte and macrophage cell lines *in vitro* (11–13). Insulin receptors are expressed on immune cells (14) and adipocytes (15), where insulin signaling intersects with inflammatory pathways, including NF-κB and MAPK cascades (16). Emerging evidence suggests that metabolic signals can directly influence chromatin organization and transcriptional activity through epigenetic remodeling. In particular, hyperinsulinemia has been shown to promote global histone acetylation through activation of the PI3K/AKT/mTOR signaling pathway, with histone H3 lysine 9 acetylation (H3K9ac) identified as a prominent marker associated with transcriptionally active chromatin. Increased H3K9ac has been linked to enhanced promoter accessibility and inflammatory gene expression in response to metabolic stress (17).

Interleukin-6 (IL-6) is an inflammatory mediator that exhibits both pro-inflammatory and anti-inflammatory activity, depending on immune context, tissue source, and duration of exposure (18). In obesity, IL-6 is recognized as a critical mediator connecting chronic inflammation with the development of insulin resistance. IL-6 promotes insulin resistance by inducing SOCS-3 expression, which inhibits insulin receptor and insulin receptor substrate-1 phosphorylation (19). IL-6 levels are markedly elevated in individuals with lipid abnormalities and insulin resistance (20); however, the mechanisms underlying sustained IL-6 overproduction by adipocytes in adipose tissue in the setting of obesity remain incompletely understood.

Because obesity is commonly associated with both metabolic endotoxemia and hyperinsulinemia, we sought to investigate whether insulin modulates LPS-induced IL-6 expression in adipocytes. Given the established crosstalk between insulin signaling and inflammatory pathways in adipocytes, we further hypothesized that insulin may potentiate LPS-induced IL-6 expression through epigenetic remodeling of the *Il6* promoter. Based on previous evidence linking hyperinsulinemia to increased histone H3 lysine 9 acetylation (H3K9ac), a marker of transcriptionally active chromatin, we focused on H3K9ac as a potential mechanistic link between metabolic dysfunction and sustained inflammatory activation. Therefore, this study aimed to determine whether insulin enhances LPS-induced IL6 expression in adipocytes and to elucidate the underlying PI3K/AKT-dependent epigenetic mechanisms involved in this regulation.

## Materials and methods

### Animal study design

This study was conducted following the National Institutes of Health guidelines for the care and use of laboratory animals. The study was reviewed and approved by the Institutional Animal Care and Ethics Committee (Approval No. RA AM 2023-023). C57BL/6 mice were purchased from the Jackson Laboratory, and the animals were bred in the Dasman Diabetes Institute animal facility and fed ad libitum on a standard chow diet. Mice were housed in a temperature-controlled room at 23 °C, with a relative humidity of 35- 40% and maintained on a 12-h dark/12-h light cycle. All experiments were performed using 8–10 weeks old male mice fed with a standard chow diet. The experimental groups were as follows:

Group 1: Control: male mice (n=3) injected with saline; Group 2: Insulin (0.5U/kg): male mice (n=3) injected intraperitoneally (IP) with insulin; Group 3: LPS (10 ng/kg): mice (n=3) injected intraperitoneally with LPS; Group 4: Insulin (0.5U/kg) +LPS (10 ng/kg): mice (n=3) injected IP with insulin, after 10 min, LPS injected. The mice were sacrificed at the end of 3 h. Blood sample and visceral adipose tissue were collected.

### Cell culture

3T3-L1 preadipocytes were purchased from the American Type Culture Collection (ATCC) and maintained in Dulbecco’s Modified Eagle’s Medium (DMEM; Gibco, Life Technologies, Grand Island, NY, USA) supplemented with 10% fetal bovine serum (FBS; Gibco, Life Technologies), 2 mM L-glutamine (Gibco, Life Technologies), and 1% penicillin–streptomycin (Gibco, Life Technologies). Cells were cultured in 6-well plates (Costar; Corning Incorporated, Washington, DC, USA) under humidified conditions at 37 °C with 5% CO_2_. Cells were sub-cultured and allowed to reach approximately 70% confluence. Then, the cells were treated with insulin (1 μg/mL; Sigma, St. Louis, MO, USA), LPS (10 ng/mL; R&D Systems, Minneapolis, MN, USA), and/or vehicle control (2% BSA). After 24 hours of treatment, the media supernatant was collected to measure IL-6 protein expression by ELISA, and cells were harvested (21) to measure *Il6* gene expression by RT-PCR. Human preadipocytes derived from subcutaneous and omental visceral adipose tissues from lean and obese individuals were obtained from ZenBio (Research Triangle Park, NC, USA; catalogue numbers: SP-F-1, OP-F-1, and OP-F-3).

### Real-time quantitative

Total RNA was extracted using the RNeasy Mini Kit (Qiagen, Valencia, CA, USA) according to the manufacturer’s instructions. Complementary DNA (cDNA) was synthesized from 1 μg of total RNA using the High-Capacity cDNA Reverse Transcription Kit (Applied Biosystems, Foster City, CA, USA) (22–25). Quantitative real-time PCR (qRT-PCR) was performed using TaqMan Gene Expression Assays (Applied Biosystems) with specific primers and probes for mouse *Il6* (Mm00446190-m1) and mouse GAPDH (Mm99999915-g1), in combination with TaqMan Gene Expression Master Mix (Applied Biosystems). Amplification and detection were carried out on a 7500 Fast Real-Time PCR System (Applied Biosystems) (26, 27). All reactions were run in triplicate under standard thermal cycling conditions. Gene expression levels were normalized to Gapdh using the 2 ^−ΔΔct^ method. Gene expression levels were expressed as fold change relative to the mean of the control group, which was set to 1.

### ELISA

The secreted IL6 levels were quantified using Elisa kit commercially available and manufactured by R&D Systems (Minneapolis, MN, USA), according to the manufacturer’s instructions. The assay’s minimum sensitivity levels of detection were 0.7 pg/ml.

### Chromatin immunoprecipitation by qRT-PCR (ChIP)

ChIP assays were performed using the SimpleChIP Plus Enzymatic Chromatin IP Kit (Cell Signaling Technology, Danvers, MA, USA) according to the manufacturer’s instructions (28). Briefly, 3T3-L1 cells were differentiated into adipocytes and treated with insulin and/or LPS. Proteins were crosslinked to DNA with 1% formaldehyde for 10 min at room temperature, followed by quenching with 125 mM glycine. Chromatin was digested with micrococcal nuclease to generate fragments of 150–900 bp. The resulting chromatin was immunoprecipitated overnight at 4 °C using antibodies specific for histone H3 lysine 9 acetylation (H3K9ac; cat. #9649, CST), histone H3 (positive control; cat. #4620, CST), or normal rabbit IgG (negative control; cat. #2729, CST), followed by incubation with Protein G magnetic beads for 2 h at 4 °C. Chromatin was eluted from the antibody–bead complexes by incubation at 65 °C for 30 min, and reverse crosslinking and protein digestion were carried out with Proteinase K for 2 h at 65 °C. DNA was purified using a spin column–based method. ChIP enrichment was determined by quantitative real-time PCR using SYBR Green chemistry and EpiTect qPCR primers (**Supplementary Table 1**) targeting transcription factor binding regions within the *Il6* promoter (**Supplementary Figure 1**). Significant association was defined as enrichment of a chromatin locus immunoprecipitated with a specific antibody relative to IgG controls (ANOVA, p < 0.05). Data are presented as mean ± SD from three independent biological experiments.

### Western blotting

3T3-L1 adipocytes treated with LPS and/or insulin were harvested and lysed in RIPA buffer for 30 min to extract total protein. Lysates were cleared up by centrifugation at 14,000×g for 10 min, and the resulting supernatants were collected. Protein concentrations were determined using the Quick Start Bradford 1× Dye Reagent Protein Assay Kit (Bio-Rad Laboratories, Hercules, CA, USA). Equal amounts of protein (20 μg) were mixed with loading buffer, denatured at 95 °C for 5 min, separated by 12% SDS–polyacrylamide gel electrophoresis, and transferred onto Immuno-Blot PVDF membranes (Bio-Rad Laboratories) by electroblotting. Membranes were blocked with 5% non-fat milk in PBS for 1 hr. and then incubated overnight at 4°C with primary antibodies against phosphorylated AKT (p-AKT; Cat. #9271, cell signaling), H3K9ac (Cat. #9649, CST), and β-actin (Cat. #4967, cell signaling) at a dilution of 1:1000. After washing three times with TBS-T, membranes were incubated for 2 h with HRP-conjugated secondary antibodies (Promega, Madison, WI, USA). Immunoreactive signals were detected using the Amersham ECL Plus Western Blotting Detection System (GE Healthcare, Buckinghamshire, UK) and visualized with the Molecular Imager^®^ VersaDoc™ MP Imaging System (Bio-Rad Laboratories).

### Statistical analysis

Data analysis was performed using GraphPad Prism software version 6.0 (La Jolla, CA, USA). Data are presented as mean ± standard deviation (SD) from at least three independent experiments/sample sets. Statistical comparisons between two groups were performed using an unpaired Student’s t-test, while comparisons among multiple groups were analyzed using one-way analysis of variance (ANOVA) followed by Tukey’s *post hoc* test. A p-value < 0.05 was considered statistically significant. Statistical significance is indicated as follows: ns, not significant; *p < 0.05; **p < 0.01; ***p < 0.001; and ****p < 0.0001.

## Results

### LPS and insulin cooperatively enhance IL-6 expression in adipocytes

**Figure 1:**
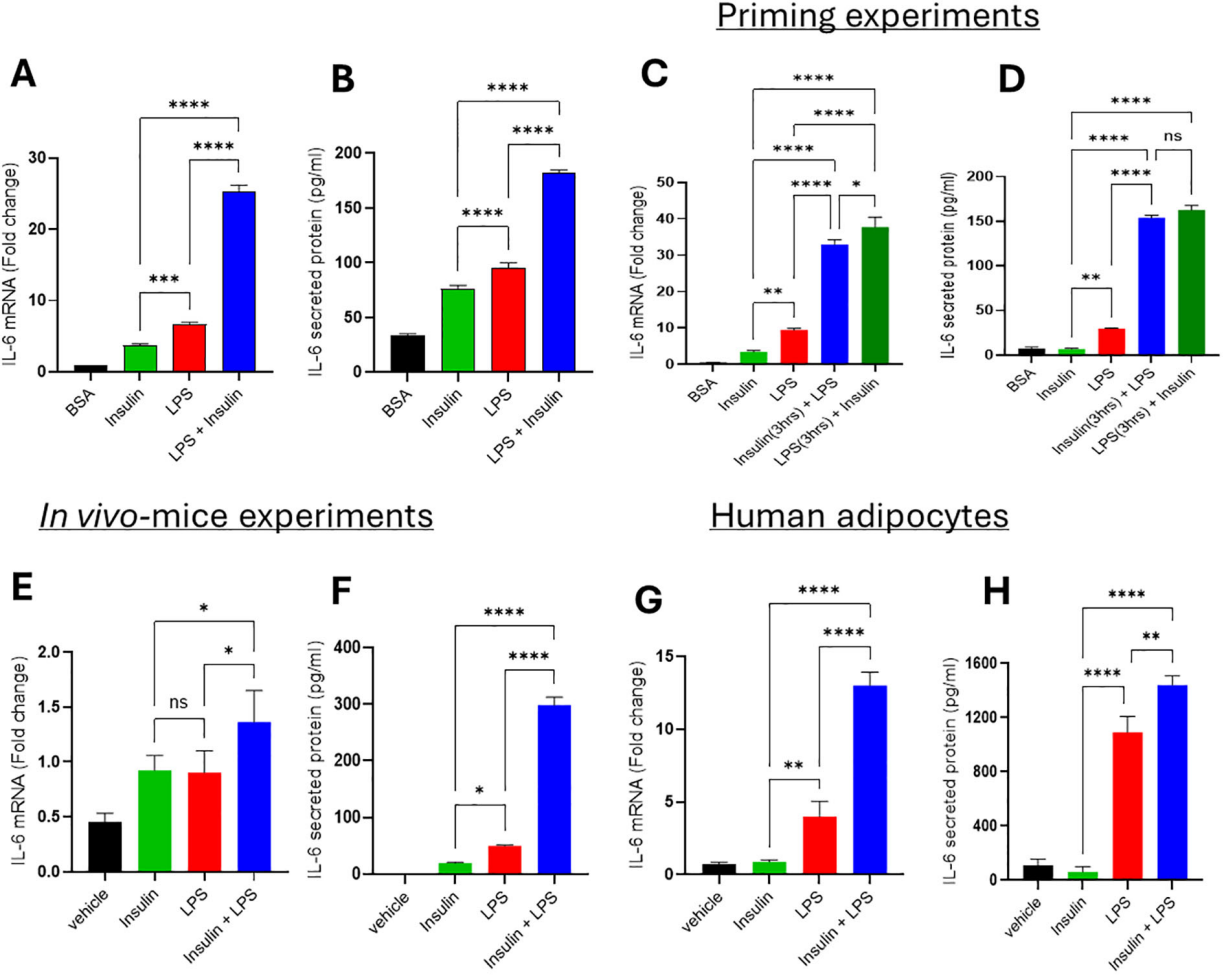
Synergistic effect of Insulin and LPS on the expression of IL-6 in adipocytes. Vehicle-treated cells served as controls, whereas experimental groups were treated with insulin (1 µg/µL) and subsequently stimulated with LPS (10 ng/mL) for 24 h. Total cellular RNA was isolated for quantification of *Il6* mRNA expression by RT-PCR, and secreted IL-6 protein levels in culture supernatants were measured by ELISA. Insulin stimulation significantly increased IL-6 mRNA **(A)** and protein secretion **(B)** in insulin-stimulated 3T3-L1 adipocytes. Priming with insulin for 3 h, followed by exposure to LPS for an additional 24 h, also induced *IL6* gene expression and protein levels **(C, D)**. Adipocytes were isolated from the VAT of C57 mice. Then, total cellular RNA was isolated from adipose tissues and used for *Il6* mRNA expression **(E)**, and IL-6 secreted protein from mouse plasma was determined using ELISA **(F)**. Human adipocytes were isolated from the VAT of a healthy participant. Then, human adipocytes were treated with LPS, insulin, or insulin + LPS, total cellular RNA was isolated and used for *Il6*mRNA expression using RT-PCR **(G)**, and IL-6 secreted protein in culture supernatants was determined using ELISA **(H)**. All data are expressed as mean ± SD values (n = 3). *p < 0.05, **p < 0.01, ***p ≤ 0.001, ****p ≤ 0.0001 is considered highly significant.

To determine whether insulin enhances LPS induced IL-6 expression in adipocytes, 3T3-L1 cells were treated with LPS or insulin alone, or in combination. Co-stimulation with insulin and LPS resulted in a marked increase in IL-6 expression at both the mRNA (25.33 ± 0.833-fold) and protein levels (181.8 ± 2.754 pg/ml) compared with either stimulus alone (**Figures 1A, B**).

To further confirm the insulin-induced priming effect, 3T3-L1 adipocytes were pretreated with insulin for 3 h before adding LPS stimulation for 24 h. Insulin priming significantly potentiated LPS-induced IL-6 production at both the mRNA and protein levels. Similarly, priming cells with LPS for 3 h followed by insulin exposure for 24 h resulted in a robust induction of *Il6* gene expression as well as protein (**Figures 1C, D**). To validate these findings *in vivo*, mice were treated with LPS, insulin, or a combination of insulin and LPS. Adipose tissue exhibited a significantly higher *Il6* gene expression following a co-treatment with insulin and LPS (1.364 ± 0.287-fold) compared with either stimulus alone, indicating a clear additive interaction (**Figure 1E**). Consistently, mice receiving insulin and LPS treatment exhibited markedly elevated plasma IL-6 protein levels compared with mice treated with either agent alone (**Figure 1F**).

To further confirm the translational relevance of this effect, primary human visceral adipocytes were treated with insulin, LPS, or insulin + LPS. Consistent with the murine data, co-stimulation with insulin and LPS induced significantly higher IL-6 expression compared with insulin or LPS alone, demonstrating a conserved synergistic response in human adipocytes (**Figures 1G, H**).

### PI3K/Akt inhibition blunts the cooperative induction of IL-6 by insulin and LPS

**Figure 2:**
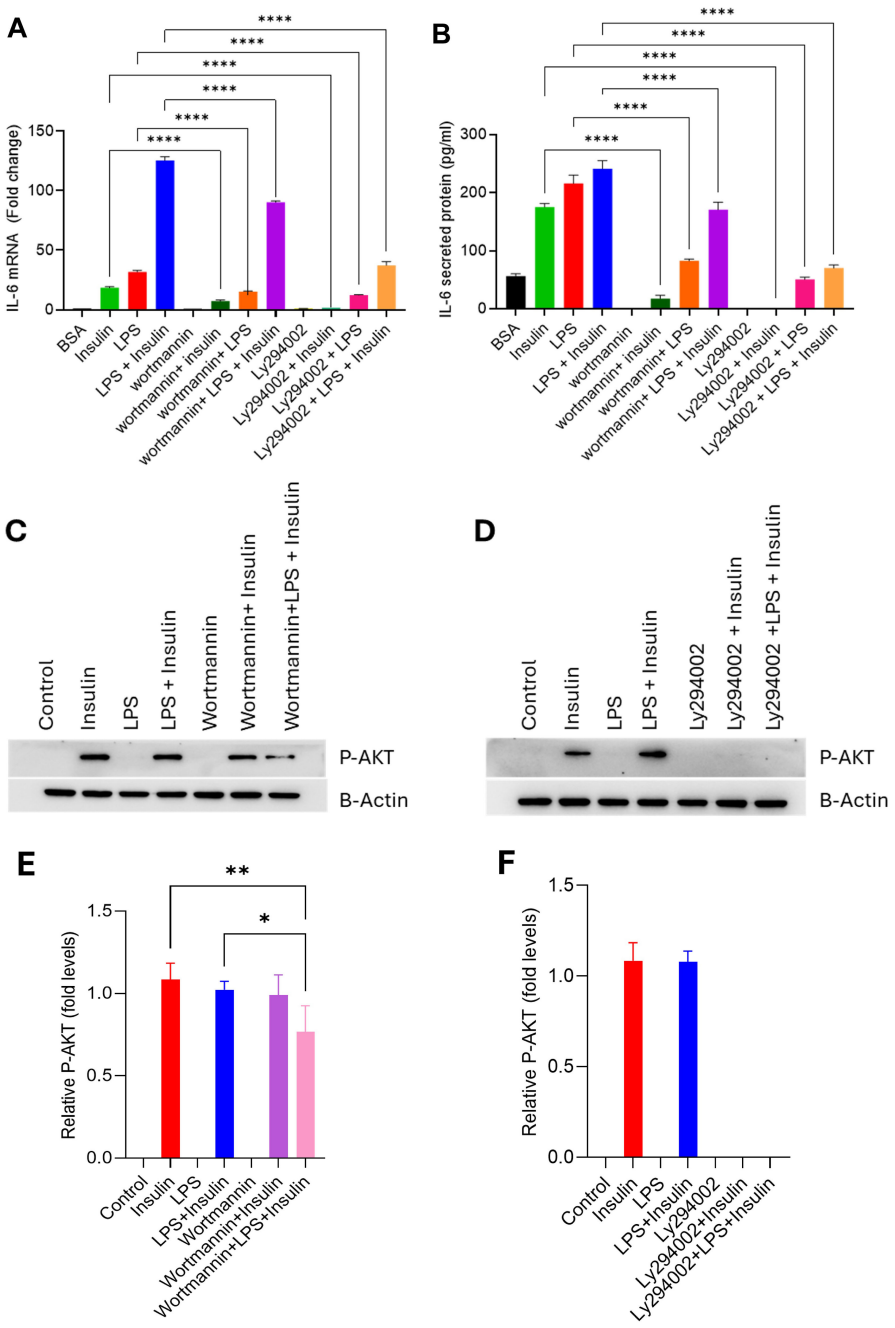
PI3K inhibition abolishes insulin-mediated enhancement of LPS-induced IL-6 expression in 3T3-L1 adipocytes. 3T3-L1 adipocytes were pre-incubated with the PI3K inhibitors LY294002 or wortmannin for 2 h, followed by stimulation with insulin, LPS, or insulin + LPS for 24 h. **(A)** RT-PCR quantified *Il6* mRNA. **(B)** Secreted IL-6 protein levels were measured by ELISA. To confirm the inhibitory effect of wortmannin on insulin signaling, Western blot analysis was performed **(C, D)**. Quantification data of Western blots are shown **(E, F)**. Data are expressed as mean ± SD (n = 3). *P <0.05; **P <0.01 ****p < 0.0001.

To determine whether PI3K/AKT activation is required for insulin-mediated potentiation of LPS-induced IL6 expression, cells were pretreated with the PI3K inhibitors LY294002 or wortmannin before insulin and LPS stimulation. Inhibition of PI3K signaling by either LY294002 or wortmannin abolished the insulin-induced enhancement of *Il6* gene expression (**Figure 2A**) and IL-6 secreted protein (**Figure 2B**). These findings demonstrate that activation of the PI3K/AKT pathway is essential for insulin-mediated amplification of LPS-induced IL-6 production. In addition, Western blotting showed enhanced AKT phosphorylation following insulin treatment, which was also observed in cells receiving combined insulin and LPS stimulation (**Figure 2C, D**). Quantification of Western blots (**Figures 2E–F**).

### Inhibition of acetylation attenuates the synergistic effect of LPS and insulin on IL-6 expression

**Figure 3:**
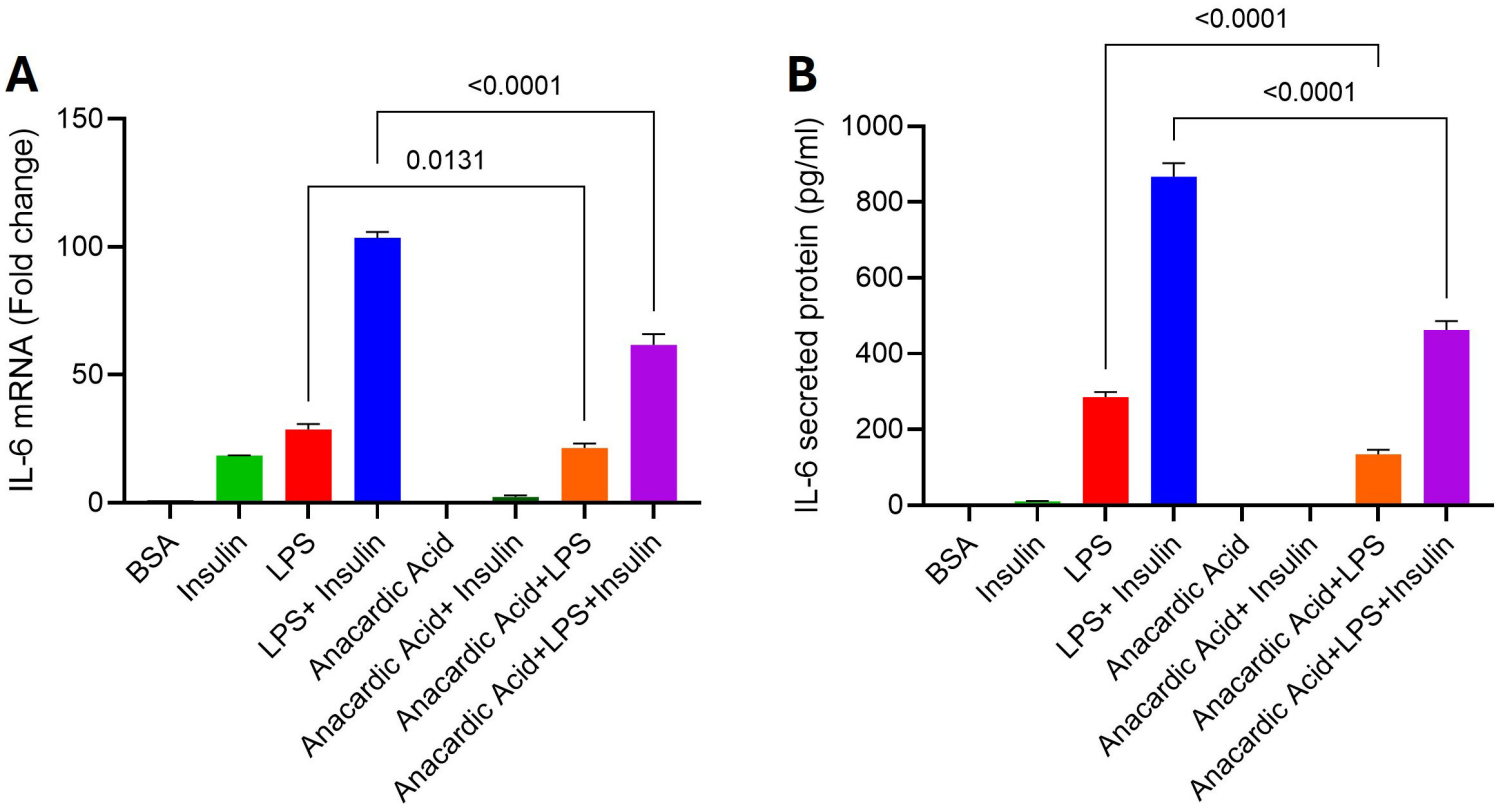
Inhibition of histone acetyltransferase activity suppresses insulin and LPS-induced IL6 expression in 3T3-L1 adipocytes. 3T3-L1 adipocytes were pre-incubated with the histone acetyltransferase (HAT) inhibitor anacardic acid (4 μM) for 2 h, followed by stimulation with insulin, LPS, or insulin + LPS for 24 h. **(A)** Total RNA was isolated, and *Il6* mRNA expression was quantified by RT-PCR, demonstrating that anacardic acid significantly reduced IL-6 expression. **(B)** Secreted IL-6 protein levels in culture supernatants were measured by ELISA. Data are expressed as mean ± SD (n = 3).

Next, we investigated whether inhibition of histone acetyltransferases (HATs) affects the synergistic effect of insulin and LPS on IL-6 secretion. 3T3-L1 adipocytes were pretreated with anacardic acid, a pharmacological HAT inhibitor, before cytokine stimulation (29). Remarkably, anacardic acid significantly reduced *Il6* mRNA expression and protein secretion (P < 0.0001) in cells treated with insulin, LPS alone or with the insulin + LPS in combination (**Figure 3A, B****).**

### Acetylation mimics the effect of insulin or LPS in promoting the synergistic induction of IL-6 expression

**Figure 4:**
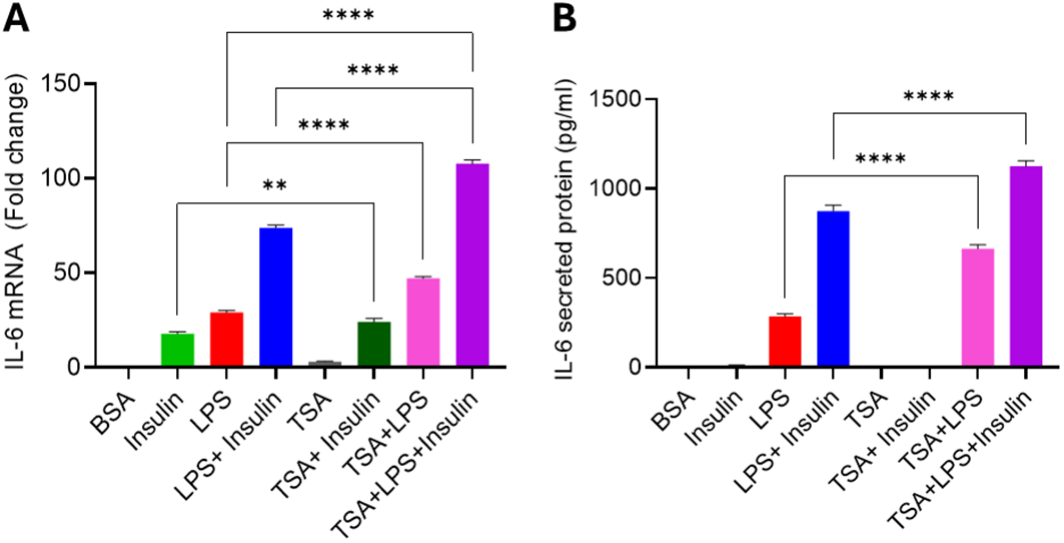
Histone deacetylase inhibition by trichostatin A (TSA) enhances insulin-LPS synergistic induction of IL6 expression. 3T3-L1 adipocytes were treated with trichostatin A (TSA; 100 nM) for 20 h, followed by stimulation with vehicle, insulin, LPS, or insulin + LPS for an additional 24 h. *Il6*mRNA expression was quantified by qRT-PCR **(A)**, and secreted IL-6 protein levels were measured in culture supernatants by ELISA **(B)**. Data were expressed as mean ± SEM. **p < 0.01, ****p < 0. 0001.

To confirm the role of histone acetylation, we examined whether pharmacological activation of histone acetyltransferases (HATs) influences the additive effect of insulin and LPS on IL-6 expression and secretion. Trichostatin A (TSA) is a histone deacetylase (HDAC) inhibitor that plays a significant role in increasing histone acetylation and gene transcription (30). To investigate whether TSA could promote *Il6* gene transcription and protein production, 3T3-L1 adipocytes were treated with TSA before stimulation with insulin, LPS, or their combination. The data show that TSA acted as a substitutive agent for either insulin or LPS in this synergy, thereby enhancing the cooperativity between insulin and LPS in the induction of *Il6* gene expression and protein production. Consistent with this observation, the additive effect of insulin and LPS on IL-6 expression and secretion was significantly increased in the presence of TSA (**Figure 4A, B**). Together, these findings suggest that histone acetylation contributes to the cooperative effect of insulin and LPS in promoting IL-6 expression and secretion.

### Insulin-induced H3K9 acetylation at NF-κB, CREB, and CREB/NF-IL6 binding loci at IL6 promoter in adipocytes

**Figure 5:**
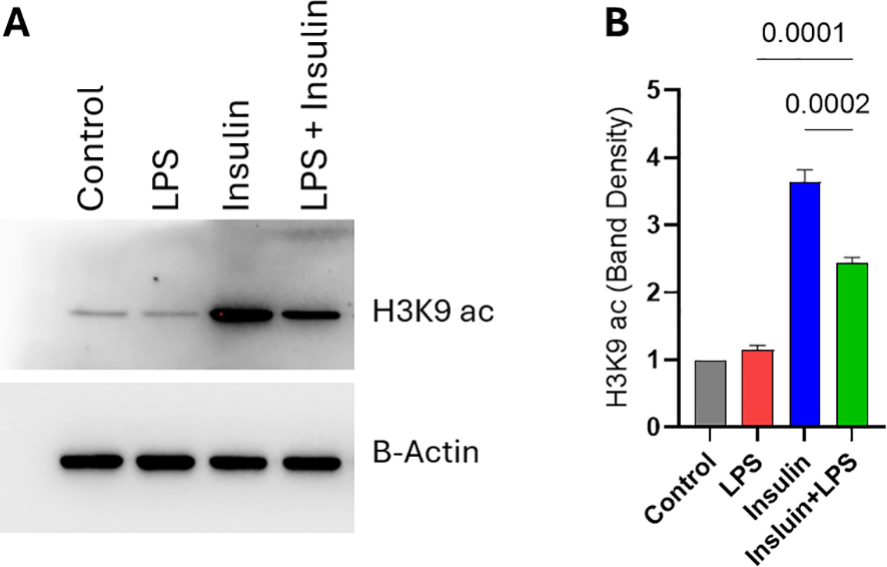
Combined treatment with insulin and LPS enhances histone acetylation. 3T3-L1 adipocytes were treated with LPS and insulin, alone or in combination. **(A)** Histone acetylation levels were assessed by Western blotting and compared between treatments and the control. **(B)** Quantification data of Western blots are shown. Data are presented as mean ± SEM. ***p < 0.001.

**Figure 6:**
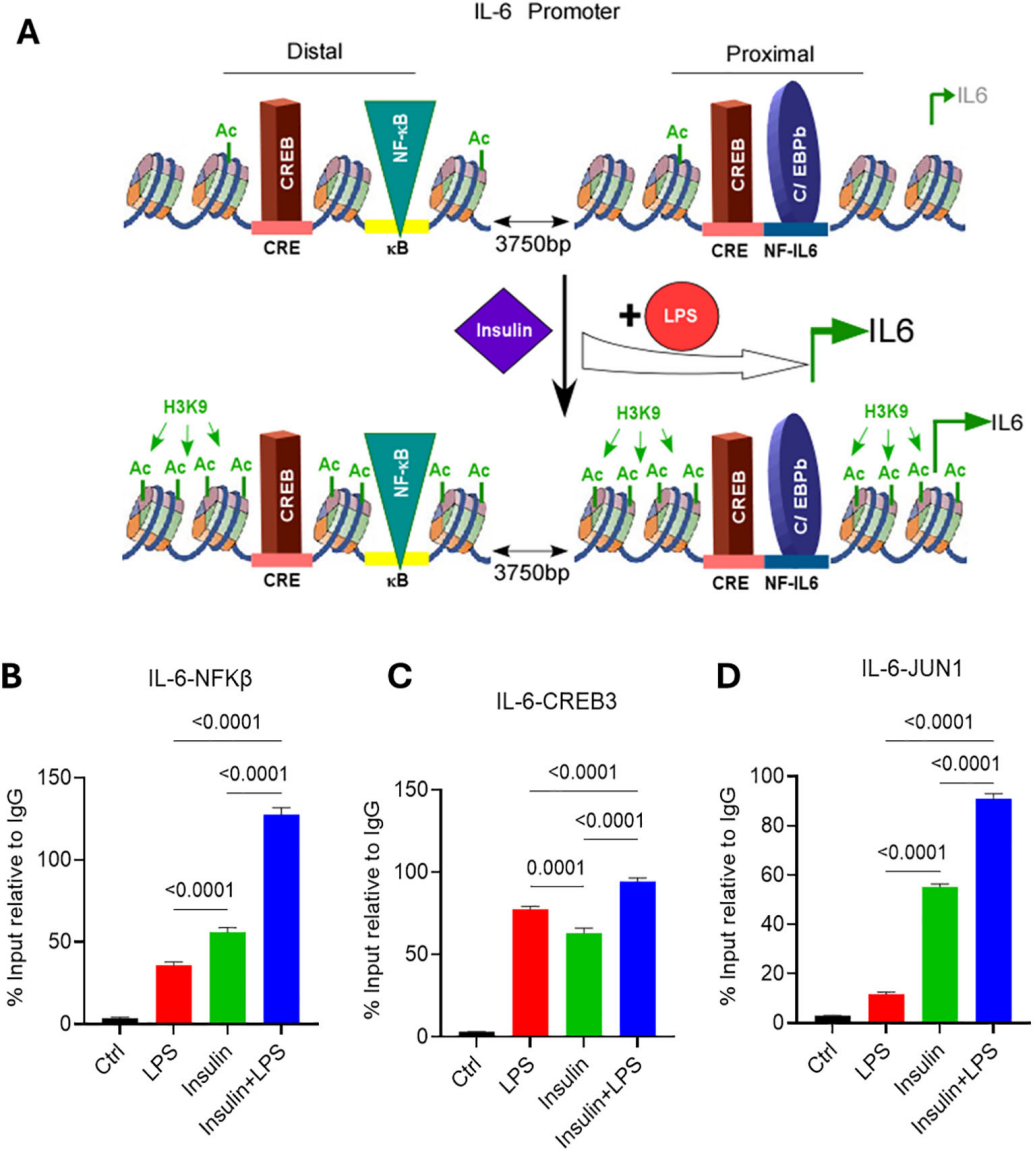
Insulin and LPS treatments enrich histone acetylation at the *Il6* promoter. 3T3-L1 adipocytes were treated with insulin and LPS, alone or in combination. **(A)** Schematic showing the proximal and distal regions of the *Il6* promoter for binding sites. The chromatin from adipocytes treated with LPS and insulin, alone or in combination, were subjected to ChIP assay using antibodies against **(B–D)** H3K9 followed by qPCR. Data are presented as mean ± SEM (n = 3), p < 0.0001.

Transcription factors promote and sustain gene transcription by recruiting histone acetyltransferases (HATs), which acetylate histones to open chromatin and increase accessibility for transcription factor binding. It is documented that insulin induced H3K9 acetylation (31). Our data showed that insulin caused hyperacetylation at histone H3K9 residues in 3T3-L1 adipocytes (**Figures 5A, B**). To assess whether insulin-induced H3K9 acetylation facilitates the binding rate of the transcription factors binding to the specific regions of the *Il6* regulatory region. Chip was performed using H3K9ac antibody following qRT-PCR at the NF-κB2 and c-Jun and CREB loci within the proximal promoter of *Il6*. Insulin or LPS stimulation of adipocytes led to an increase in H3K9ac levels at NF-κB, CREB, and CREB/NF-IL6 binding regions examined in the *Il6* promoter. Co-treatment of insulin and LPS further enhanced H3K9ac at these loci of the *Il6* promoter (**Figures 6A–D**). Taken together, these results indicate that the acetylation of H3K9 is strongly associated with this synergy between insulin and LPS for *Il6* gene transcription.

## Discussion

Chronic low-grade inflammation within adipose tissue is a hallmark of obesity and a major contributor to the development of insulin resistance and type 2 diabetes (4). Among inflammatory mediators, IL-6 plays a central role in linking metabolic dysfunction with systemic inflammation (32). However, IL-6 exerts both pro-inflammatory and anti-inflammatory functions depending on the cellular source, tissue context, signaling mode, and duration of exposure. While transient IL-6 signaling may contribute to immune regulation and metabolic adaptation, chronic elevation of IL-6 in obesity is strongly associated with adipose tissue inflammation, insulin resistance, and metabolic dysfunction (33, 34). In the present study, we demonstrate that insulin markedly potentiates LPS induced IL-6 expression in adipocytes through a mechanism involving PI3K-AKT signaling and epigenetic remodeling of the *Il6* promoter. Specifically, insulin promotes H3K9ac, which enhances the recruitment of transcription factors including NF-κB, CREB, and CREB/NF-IL6 at the distal and proximal *Il6* promoter regions. These findings provide mechanistic insight into how metabolic and inflammatory signals converge at the chromatin level to amplify inflammatory gene transcription in adipocytes.

Previous studies have shown that LPS, a component of gram-negative bacterial cell walls, is a potent inducer of inflammatory cytokines in adipocytes and immune cells through activation of Toll-like receptor 4 (TLR4) and downstream NF-κB signaling pathways (35). Moreover, metabolic endotoxemia, characterized by elevated circulating LPS levels, has been reported in obesity and has been implicated in promoting adipose tissue inflammation and insulin resistance (36). While these studies established LPS as an important trigger of inflammatory signaling in metabolic tissues, the influence of metabolic hormones such as insulin on LPS-driven inflammatory responses has remained less well defined. These observations were further extended by demonstrating that insulin can function as a priming signal that amplifies inflammatory responses in adipocytes. Previous reports have suggested that insulin may exert either pro- or anti-inflammatory effects depending on the cellular context and signaling environment (37). In macrophages and endothelial cells, insulin has been reported to suppress certain inflammatory pathways, whereas other studies have shown that hyperinsulinemia can promote inflammatory signaling in metabolic tissues (38–40). Our findings support the latter concept by demonstrating that insulin significantly enhances LPS induced IL-6 expression. Importantly, the synergistic effect observed in our study was greater than the additive effect of the two stimuli alone, indicating a true cooperative interaction between insulin signaling and innate immune activation. This observation suggests that under conditions of hyperinsulinemia, such as those commonly observed in obesity and early type 2 diabetes, adipocytes may become more responsive to inflammatory stimuli.

A key mechanistic insight from our study is the identification of the PI3K-AKT pathway as a critical mediator of insulin dependent amplification of IL-6 expression. The PI3K-AKT signaling cascade represents the canonical downstream pathway of the insulin receptor and is well known for regulating glucose uptake, lipid metabolism, and cell survival (41, 42). However, emerging evidence suggests that this pathway can also influence inflammatory gene expression (31, 43). Consistent with this concept, our data show that pharmacological inhibition of PI3K suppressed the insulin-mediated potentiation of LPS induced IL-6 expression, indicating that activation of this pathway is required for the cooperative inflammatory response. These findings suggest that PI3K-AKT signaling may serve as an important molecular bridge between metabolic and inflammatory pathways in adipocytes (44, 45). Another major contribution of this study is the demonstration that epigenetic remodeling plays a central role in the synergistic induction of IL-6 expression. Histone acetylation is widely recognized as a key epigenetic mechanism that regulates chromatin accessibility and transcriptional activation (46, 47). Inflammatory stimuli such as LPS can induce transcription factors binding at promoters of pro-inflammatory genes, thereby facilitating transcription factor binding (48). However, relatively little is known about how metabolic signals influence these epigenetic modifications. Our results reveal that insulin increases H3K9 acetylation at regulatory regions of the *Il6* promoter in a PI3K-dependent manner, thereby enhancing chromatin accessibility and transcription factor recruitment. This observation provides direct evidence that metabolic signaling can modulate inflammatory gene expression through epigenetic mechanisms. Consistent with this model, pharmacological inhibition of histone acetyl transferase activity with anacardic acid significantly reduced IL-6 expression induced by insulin and by combined insulin and LPS treatment. Conversely, TSA induced hyperacetylation enhanced IL-6 expression and partially mimicked the effect of insulin in the cooperative response. These findings strongly support the conclusion that histone acetylation is a key regulatory step in the integration of metabolic and inflammatory signaling pathways. ChIP analyses further demonstrate that increased H3K9 acetylation is associated with enhanced binding of NF-κB, CREB, and CREB/NF-IL6 to the *Il6* promoter following combined insulin and LPS stimulation. NF-κB is a well-established regulator of inflammatory gene expression, and its activation downstream of TLR signaling is critical for cytokine production. CREB and AP-1 family transcription factors, including JUN proteins, have also been implicated in the regulation of inflammatory and metabolic genes (49, 50). Together, these findings support the concept that insulin-induced chromatin remodeling creates a permissive transcriptional environment that facilitates Il6 gene transcription. These findings have important implications for the pathophysiology of obesity and diabetes. In individuals with obesity, circulating insulin levels are frequently elevated due to insulin resistance, while metabolic endotoxemia resulting from increased intestinal permeability contributes to higher systemic levels of LPS. Our data suggest that these two factors may cooperate to amplify inflammatory signaling within adipose tissue. By enhancing chromatin accessibility and transcription factor recruitment at the *Il6* promoter, insulin may prime adipocytes to produce exaggerated inflammatory responses to microbial and metabolic stimuli. Sustained IL-6 production can perpetuate chronic low-grade inflammation, impair insulin signaling in peripheral tissues, and accelerate the progression of metabolic disease. IL-6 has been implicated in the development of insulin resistance through multiple mechanisms, including activation of SOCS proteins that inhibit insulin receptor signaling and modulation of hepatic glucose metabolism. Elevated circulating IL-6 levels are consistently observed in patients with obesity and type 2 diabetes and strongly correlate with markers of metabolic dysfunction. Therefore, the mechanism identified in our study, whereby insulin enhances *Il6* expression through PI3K–AKT-dependent epigenetic remodeling, may represent an important molecular link between hyperinsulinemia and chronic inflammatory activation during the progression of metabolic disease.

Importantly, our findings also identify a potential therapeutic strategy to disrupt the pathogenic interaction between hyperinsulinemia and inflammation in obesity. The observation that insulin-driven IL-6 amplification depends on PI3K–AKT signaling and histone acetylation suggests that targeting this pathway may attenuate adipose tissue inflammation without completely suppressing basal immune function. In particular, modulation of chromatin remodeling and histone acetylation may represent a novel approach to selectively reduce pro-inflammatory cytokine expression in adipocytes. Our findings with anacardic acid and TSA further support the concept that epigenetic regulators play a critical role in controlling inflammatory gene transcription in response to metabolic signals. These observations raise the possibility that pharmacological targeting of histone acetyltransferases, chromatin accessibility, or downstream transcriptional complexes could limit the persistent inflammatory state associated with obesity and insulin resistance. In addition, therapeutic strategies aimed at reducing metabolic endotoxemia or interrupting insulin-mediated inflammatory priming may help break the feed-forward cycle linking gut-derived inflammatory stimuli, hyperinsulinemia, and adipose tissue dysfunction.

Several limitations of this study should also be acknowledged. First, some *in vitro* experiments utilized relatively high insulin concentrations that may not fully reflect physiological conditions, although such concentrations are commonly used to model hyperinsulinemic states in mechanistic studies. Second, the sample size in the *in vivo* experiments was relatively small, which may limit the generalizability of the findings. Third, the current study employed an acute inflammatory stimulation model rather than a chronic obesity or insulin resistance model. Therefore, additional studies using long-term *in vivo* models of metabolic disease will be necessary to confirm the physiological relevance of the identified mechanism and to determine its contribution to chronic adipose tissue inflammation during obesity progression.

In summary, our findings reveal a previously underappreciated mechanism through which insulin amplifies inflammatory signaling in adipocytes. By activating the PI3K–AKT pathway and promoting H3K9 acetylation at the *Il6* promoter, insulin enhances transcription factor recruitment and synergistically increases IL-6 expression in response to LPS. This epigenetic mechanism provides a molecular link between hyperinsulinemia, metabolic endotoxemia, and chronic inflammation in obesity and diabetes. More importantly, our study highlights the PI3K–AKT–chromatin remodeling axis as a promising therapeutic target for limiting adipose tissue inflammation and mitigating metabolic complications associated with insulin resistance and type 2 diabetes. Future *in vivo* and translational studies will be important to determine whether targeting this inflammatory epigenetic program can improve metabolic health and reduce obesity-associated disease progression.

## Data Availability

The original contributions presented in the study are included in the article. Further inquiries can be directed to the corresponding authors.
