# Supplementary material for: Insulin potentiates lipopolysaccharide-induced IL-6 expression through epigenetic remodeling in adipocytes: in vitro and in vivo mechanistic study
Source: Front Immunol. 2026 Jul 8;17:1840850. doi: 10.3389/fimmu.2026.1840850 (PMC13388075; doi:10.3389/fimmu.2026.1840850)
Supplement: Supplementary file 1 [file DataSheet1.pdf]

Supplementary Table S1 and Figure S1

Table S1: Primer Sequence

Target Region	Primer Direction	Sequence (5′ → 3′)
IL6-NFκB	Forward (F)	GCTGAGGTTT CCTAATGGTGA
	Reverse (R)	CCGAGCTGGTT GGTGATAAG
IL6-Creb	Forward (F)	ATGGCTCTGAACCGAGAGAA
	Reverse (R)	CCTCTCTCTGTGGGGTTGAT
IL6-C/EBPb	Forward (F)	TAGGGCTAGCCTCAAGGATG
	Reverse (R)	GGGGCTGATTGGAAACCTTA

Figure S1:

IL-6 Distal Promoter

actacagcagagactttattcagaggaactaagacattagcaaataactg
ataaaaaggaagggaggtaaaatacatcacagtcaggccaaaagaatccc
ctggttttgacacaacatgtgatgctttcaactagttcctactaccttaa
ttttaagggaaaattgatactttgcatgcttagtttctgctc**atggctct**
CREB
gaaccgagagaactgagatctttggagaagccaaagtggtttaagtgact
taccagaataaatgagctggtttctctta**gctgaggtttcctaatggtga**
agcacaagtagcagccactgaaa**atcaaccccacagagagagg**caagcac
NFκB
agaaagtcagcccaagggatcttcccggaccggggagagttcaggatatc
aactcgatgt**cttatcaccaaccagctcgg**agaggccatcttatcgacac
ttagggatcagagcactctcattaagataagacagaaatgtgtagcaggt

IL-6 Proximal Promoter

taaaacattgtgaatttcagttttctttcccatcaagacatgctcaagtg
ctgagtcacttttaaagaaaaaaaagaagagtgctcatgcttct**tagggc**
tagcctcaaggatgacttaagcacactttccccttcctagttgtgattct
CREB C/EBPb
ttcgatgctaaacgacgtcacattgtgcaatcttaa**taaggtttccaatc**
agccccacccactctggccccaccccaccctccaacaaagattttatc
aaatgtgggattttcccatgagtctcaaaattagagagttgactcctaat
AAATATGAGACTGGGGATGTCTGTAGCTCATTCTGCTCTGGAGCCCACCA
Translation start Site
AGAACGATAGTCAATTCCAGAAACCGCT**ATG**

Figure S1: The proximal and distal promoters' sequences of IL-6. The genomic sequence shows the consensus side binding for the transcription factors CERB and C/EBPb at the proximal promoter, named CRE and NF-IL6 sequences. The transcription factors binding to their prospective consensus sites are CREB and NFkB. The primers used in this study are the forward (underlined black font), and reverse (underlined red font), each pair of primers flanking the appreciated transcription binding side. The 5'-untraslated sequence (upper case letter) and the translation start side (ATG) are shown. The Table shows the primer sequence 5'to 3' direction. In our previous publication we showed that these transcription factors are binding to their prospective consensus sites. https://doi.org/10.3390/ijms25126776.
